# Postoperative spinal epidural hematoma in a biportal endoscopic spine surgery

**DOI:** 10.1097/MD.0000000000024685

**Published:** 2021-02-12

**Authors:** Dong Ki Ahn, Jung Soo Lee, Won Shik Shin, San Kim, Jin Jung

**Affiliations:** Seoul Sacred Heart General Hospital Orthopedic Surgery Department.

**Keywords:** biportal endoscopic spine surgery, postoperative spinal epidural hematoma

## Abstract

Biportal endoscopic spine surgery (BESS) is extending its application to most kind of spine surgeries. Postoperative spinal epidural hematoma (POSEH) is one of the major concerns of this emerging technique. Through this study we aim to investigate the incidence of POSEH in BESS comparing to a conventional spine surgery (CSS).

The patients who underwent a non-fusion decompressive spine surgery due to degenerative lumbar spinal stenosis (LSS) or herniated lumbar disc (HLD) or both between January 2015 and March 2019 were reviewed retrospectively. The incidence of clinical POSEH that demanded a revision surgery for hematoma evacuation was compared between CSS and BESS. As a second endpoint, the morphometric degree of POSEH was compared between the two groups. The maximal compression of cauda equina by POSEH was measured by 4 grade scale at the T2 axial image and the neurological state was evaluated by 5 grade scale. The indication of hematoma evacuation was more than hG3 with more than nG1. As a subgroup analysis, risk factors of POSEH in BESS were investigated.

The 2 groups were homogenous in age, sex, number and level of operated segments. There was significant difference in the incidence of symptomatic POSEH as 2/142 (1.4%) in CSS and 8/95 (8.4%) in BESS (*P* = .016). The radiological thecal sac compression by hematoma was hG1 65 (61.3%), hG2 35 (33.0%), hG3 5 (4.7%), hG4 1 (0.9%) cases in CSS and hG1 33 (39.8%), hG2 25 (30.1%), hG3 22 (26.5%), hG4 3 cases (3.6%) in BESS. The difference was significant (*P* < .001). In BESS subgroup analysis, the risk factor of high grade POSEH was bilateral laminectomy (OR = 8.893, *P* = .023).

The incidence of clinical and morphometric POSEH was higher in BESS. In BESS, POSEH developed more frequently in bilateral laminectomy than unilateral laminectomy.

## 1 Introduction

Minimal invasive surgical options have many merits,^[1,2]^ however, there were also limitations of their indications due to limited view and working angle. As far as I know, the biportal endoscopic spine surgery (BESS) is one of the most versatile tools that can be applied to various spinal diseases almost same as an open conventional spine surgery (CSS).^[3–7]^ But there are many concerns on its safety.^[8,9]^ Postoperative epidural hematoma (POSEH) is one of the complications that are considered developing more often in BESS than CSS. In CSS the incidence and risk factors of POSEH have been reported inconsistently. Besides, there are more issues in BESS. We presumed that intra-operative bleeding control is difficult due to various reasons while performing BESS. We compared the clinical and morphometric difference in terms of POSEH and also investigated the risk factors of POSEH in BESS group independently.

## 2 Methods

This study received the approval of public institutional review board (POI-202005–21-006) and the written informed consent from the patients was exempted. It was a retrospective case-controlled study in which we reviewed the medical records and radiological exams of the patients those who received a decompression surgery without fusion due to a degenerative lumbar spinal disease between January 2015 and March 2019. Before March 2017, all patients received CSS. From then on, we treated all patients with BESS. Those who had a revision surgery, foraminal stenosis, extraforaminal herniated disc were excluded.

### 2.1 Operation methods

In CSS, conventional midline approach was used. In case of bilateral laminectomy, midline spinous process was excised as much as necessary. An electric coagulator and a bipolar coagulator (Vellylab^TM^, Medtronic, Minneapolis USA) were used for the muscle dissection and bleeding control. A bone wax (Ethicon^TM.^ Los Frailes Industrial Park, USA) was used for the hemostasis of bone surface. In BESS, unilateral two portals (one for endoscopy and the other for instrumental working) were made. Radiofrequency coagulator (ArthroCare^TM^ Austin, Texas USA) was used for soft tissue hemostasis and same type bone wax was used for bone surface hemostasis. Saline pressure was fixed at 50 mm Hg. None of the thrombin or fibrinogen containing haemostatic agents was used in both groups. A vacuum suction drain was placed and connected to negative pressure bag 120 ± 30 mm Hg (Ez-VAC^,^ EZ Medisys, Koyang, Korea) in all cases of both groups and it was removed at day 2 after the operation.

### 2.2 Data analysis

**Figure 1:**
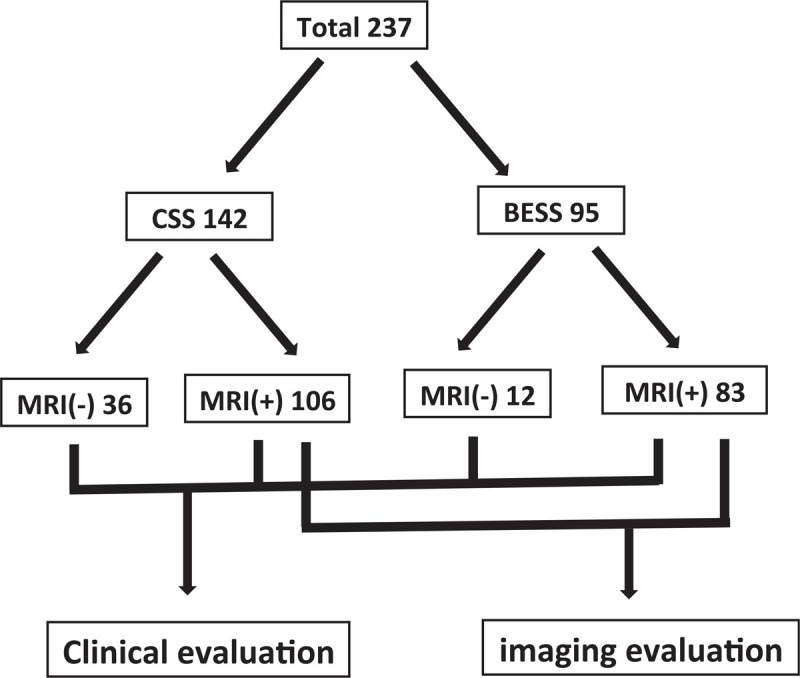
The flow diagram of the subjects.

**Figure 2:**
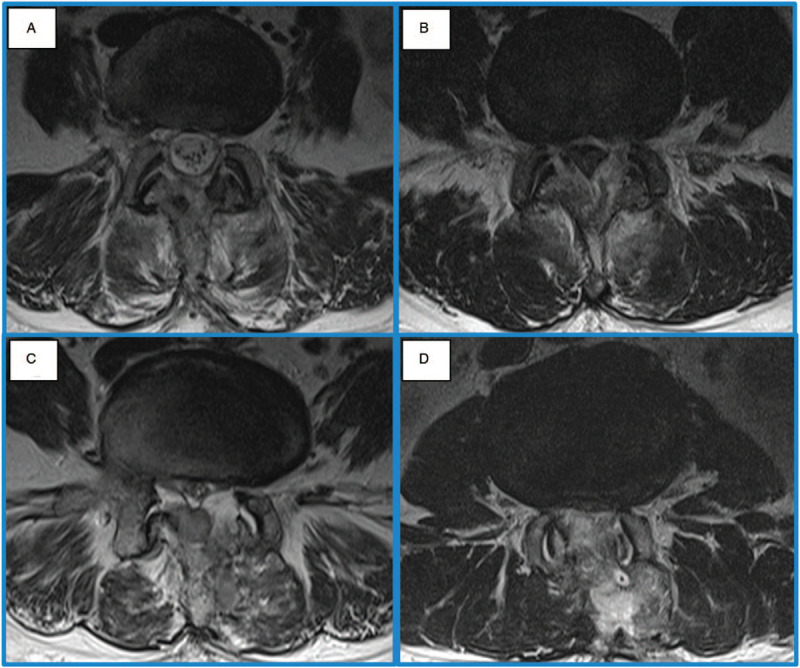
The grading system of thecal sac compression by epidural hematoma in T2 MRI axial images. (A) Grade 1; that stands for the thecal sac compression less than a quarter. (B) Grade 2; between a quarter and a half. (C) Grade 3; between a half and three quarters. (D) Grade 4; over three quarters.

The demographic data, coagulation related data were analyzed to prove the homogeneity. There were 142 patients in CSS and 95 patients in BESS. Among them, 106/142 cases (75%) of CSS and 83/95 cases (87%) of BESS had postoperative magnetic resonance imaging (MRI) exam. Those who did not have a postoperative MRI were included in the clinical evaluation and excluded in the imaging evaluation (Fig. 1). The routine postoperative MRI was taken at the 7th ± 1 day. However, if there were urgent symptoms that suggested POSEH, MRI was taken immediately when we discovered the symptoms. On the postoperative MRI, the maximal compression site of thecal sac by POSEH was measured by 4 grade scale at a T2 axial image as follows; hG1: thecal sac compression less than 1 quarter, hG2: between one quarter and half, hG3: between half and three quarters, hG4: more than 3 quarters obstruction (Fig. 2). The MRIs were interpreted by an orthopedic surgeon who was blinded to the study design. The neurological state was evaluated by 5 grade scale as follows; nG0: normal, nG1: unilateral or bilateral leg pain and numbness, nG2: unilateral motor weakness, nG3: bilateral motor weakness, nG4: cauda equina syndrome. The patients who had a neurological deficit that was aggravated comparing to the preoperative state or the ones not improved as much as expected and the ones who had an MRI compatible with POSEH (≥hG3) underwent a revision surgery for hematoma evacuation. Among the patients who had hematoma evacuation, only those who showed immediate improvement were confirmed as POSEH. The revision surgeries in CSS were performed under a general anesthesia while those in BESS were done under local anesthesia with BESS except a case. The incidence of clinical POSEH and morphometric POSEH were compared. And as a subgroup analysis, risk factors of POSEH in BESS were investigated.

In statistical analysis, parametric variables were analyzed with student T test and nonparametric variables were done with Fisher exact test and Chi-Squared test. In cases that expected frequency was less than 5, likelihood ratio test was applied. The morphometric comparison of POSEH was done with Mann–Whitney *U* test. In subgroup analysis, the risk factor was analyzed with multivariable logistic regression test. SPSS for Windows software package (ver. 16.0; SPSS Inc., Chicago, IL, USA) was used.

## 3 Results

**Figure 3:**
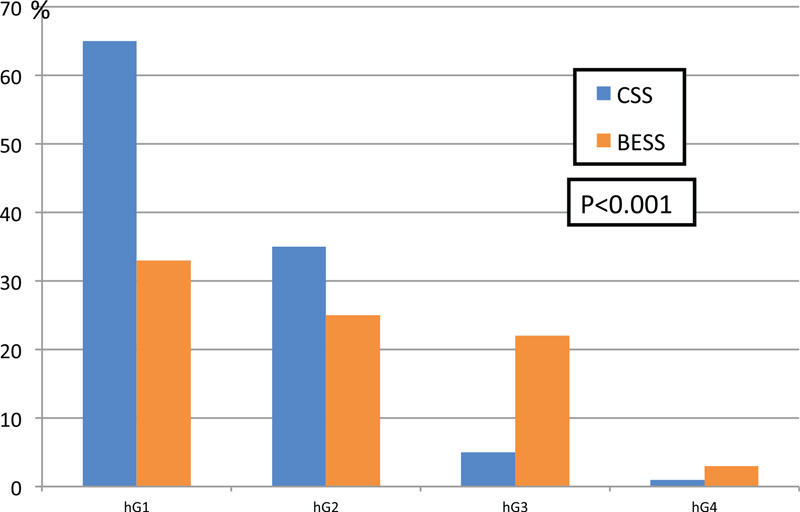
The difference of Morphometric POSEH between the 2 groups.

Two groups were homogenous in age, sex, number and level of operated segments, however, there was a heterogeneity in diagnosis. The ratio of HNP was higher in CSS (Table 1). There was significant difference in the incidence of clinical POSEH as 2/142 (1.4%) in CSS and 8/95 (8.4%) in BESS (*P* = .016, OR = 6.962). The grade of neurological deficit in CSS was nG1 for all 2 cases and that in BESS was nG1 for 6, nG2 for 1 and nG3 for 1. The 2 cases who had nG2 and nG3 received MRI exam at day 3 and 2 respectively due to the clinical urgency. The radiological thecal sac compression by hematoma, in other words morphometric POSEH was hG1 65 (61.3%), hG2 35 (33.0%), hG3 5 (4.7%), hG4 1 (0.9%) cases in CSS and hG1 33 (39.8%), hG2 25 (30.1%), hG3 22 (26.5%), hG4 3 (3.6%) cases in BESS. The difference was significant (*P* < .001) (Table 1) (Fig. 3).

**Table 1:** Demographic data and incidence of POSEH^∗^.

	CSS^†^	BESS^‡^	*P* value
Age	62.2 *±* 12.8	66.2 ± 11.5	.164
Sex (F/M)^§^	77/65	55/40	.596
No^||^ of segments	1.3 *±* 0.6	1.2 *±* 0.4	.129
Diagnosis Stenosis/HLD^¶^/both	60/70/12	60/21/14	<.001
Antiplatelet drug +/-	12/130	11/85	.254
Platelet	253,190 ± 59,129	255,730 ± 66,480	.759
PFA-Epi^∗∗^	178.0 ± 79.4	160.2 ± 81.0	.096
PT^††^	10.5+1.1	10.7 + 0.7	.440
aPTT^‡‡^	28.4 ± 4.2	27.4 ± 2.5	.323
POSEH, clinical (+/-)	2/142 (1.4%)	8/95 (8.4%)	.016
nG1	2 (100%)	6 (75%)	OR^§§^: 6.962
nG2	0	1 (12.5%)	
nG3	0	1 (12.5%)	
nG4	0	0 (0%)	
POSEH, morphometrical			<.001
hG1	65 (61.3%)	33 (39.8%)	
hG2	35 (33.0%)	25 (30.1%)	
hG3	5 (4.7%)	22 (26.5%)	
hG4	1 (0.9%)	3 (3.6%)	
Total	106 (100%)	83 (1005)	

∗ Postoperative spinal epidural hematoma.

† Conventional spine surgery.

‡ Biportal endoscopic spine surgery.

§ Female/Male.

|| Number.

¶ Herniated lumbar disc.

∗∗ Platelet function analysis-epinephrine.

†† Prothrombin time.

‡‡ Activated partial thromboplastin time.

§§ Odds ratio.

The risk factors of POSEH were analyzed for the all subjects. In clinical POSEH, only BESS was the risk factor through a multivariable analysis (*P* = .032, OR = 4.998) (Table 2). In morphometric POSEH the subjects were divided into small (G1∼2) and large (G3∼4). BESS was also the only significant risk factor through a multivariable analysis (*P* < .001, OR = 4.998) (Table 3).

**Table 2:** Risk factors for clinical POSEH^∗^, univariable and multivariable.

			*P* value	
POSEH, clinical	+ (10)	− (226)	univariable	multivariable
Operation methods (CSS/BESS)^†^	2/8	140/86	0.008	.032
				OR^‡‡^: 4.998
Age	64.7 *±* 9.5	63.7 *±* 12.6	0.798	
Sex (F/M)^‡^	6/5	126/100	1.000	
Diagnosis Stenosis/HLD^§^/both	8/1/1	111/90/25	0.098	.473
No^||^ of segment	1.3 *±* 0.6	1.2 ± 0.5	0.732	
Unilateral/bilateral	1/9	90/136	0.035	.320
Platelet	242,360 ± 74,329	254,780 ± 61,533	0.518	
PFA-Epi^¶^	190.4 ± 86.5	170.0 ± 80.0	0.411	
PT^∗∗^	10.5 ± 0.6	10.6 ± 0.6	0.458	
aPTT^††^	28.3 ± 2.3	28.1 ± 3.2	0.832	

∗ Postoperative spinal epidural hematoma.

† Conventional spine surgery/Biportal endoscopic spine surgery.

‡ Female/Male.

§ Herniated lumbar disc.

|| Number.

¶ Platelet function analysis-epinephrine.

∗∗ Prothrombin time.

†† Activated partial thromboplastin time.

‡‡ Odds ratio.

**Table 3:** Risk factors for morphometrical POSEH in whole subjects.

			*P* value	
POSEH^∗^ morphometrical	Small (G1∼2) (157)	Large (G3∼4) (32)	univariable	multivariable
Operation methods (CSS/BESS)^†^	99/58	7/25	**<**.001	<.001
				OR^‡‡^: 4.983
Age	63.1 ± 13.0	65.7 ± 11.7	.293	
Sex (F/M)^‡^	88/69	21/11	.431	
Diagnosis Stenosis/HNP^§^/both	66/69/22	24/5/3	.004	.116
No^||^ of segment	1.2 ± 0.5	1.2 ± 0.5	.713	
Unilateral/bilateral	69/88	5/27	.001	.152
Platelet	258,410 ± 65,211	246,740 ± 51,997	.349	
PFA-Epi^¶^	170.3 ± 81.2	194.7 ± 86.2	.132	
PT^∗∗^	10.6 ± 0.6	10.7 ± 0.6	.605	
aPTT^††^	28.2 ± 3.3	28.0 ± 2.7	.807	

∗ Postoperative spinal epidural hematoma.

† Conventional spine surgery/Biportal endoscopic spine surgery.

‡ Female/Male.

§ Herniated nucleus pulposus.

|| Number.

¶ Platelet function analysis-epinephrine.

∗∗ Prothrombin time.

†† Activated partial thromboplastin time.

‡‡ Odds ratio.

In subgroup analysis of BESS, there was no significant risk factor of clinical POSEH (Table 4) and unilateral/bilateral laminectomy was significant (*P* = .023) and PFA-Epi was close to the significant level of a risk factor in morphometrical POSEH multivariable analysis (*P* = .086) (Table 5).

**Table 4:** Risk factors for clinical POSEH in BESS group.

			*P* value	
POSEH^∗^, clinical	+ (8)	− (87)	univariable	multivariable
Age	67.2 ± 8.2	66.1 ± 11.8	.783	
Sex (F/M)^†^	4/4	51/36	.486	
Diagnosis Stenosis/HNP^‡^/both	7/0/1	53/21/13	.212	
No^§^ of segment	1.3 ± 0.7	1.1 ± 0.4	.168	
Unilateral/bilateral	0/8	21/66	.095	.998
platelet	247,110 ± 79,791	256,630 ± 65,420	.685	
PFA-Epi^||^	210.8 ± 82.2	155.0 ± 79.5	.049	.095
PT^¶^	10.7 ± 0.5	10.7 ± 0.7	.715	
aPTT^∗∗^	28.7 ± 2.3	27.2 ± 2.5	.096	.160

∗ Postoperative spinal epidural hematoma.

† Female/Male.

‡ Herniated nucleus pulposus.

§ Number.

|| Platelet function analysis-epinephrine.

¶ Prothrombin time.

∗∗ Activated partial thromboplastin time.

**Table 5:** Risk factors for morphomeric POSEH in BESS group.

			*P* value	
POSEH^∗^, morphological	Small (G1∼2) (58)	Large (G3∼4) (25)	univariable	Multivariable
Age	65.3 ± 11.7	69.6 ± 9.1	.107	
Sex (F/M)^†^	35/23	15/10	1.000	
Diagnosis Stenosis/HNP^‡^/both	29/18/11	21/1/3	.004	.281
No^§^ of segment	1.1 ± 0.4	1.2 ± 0.5	.390	
Unilateral/bilateral	18/40	1/24	.009	.047, OR^††^: 8.893
Platelet	260980 ± 72536	246360 ± 50987	.363	
PFA-Epi^||^	149.3 ± 77.0	186.7 ± 82.6	.050	.086
PT^¶^	10.7 ± 0.7	10.7 ± 0.5	.675	
aPTT^∗∗^	27.1 ± 2.7	27.8 ± 2.4	.242	

∗ Postoperative spinal epidural hematoma.

† Female/Male.

‡ Herniated nucleus pulposus.

§ Number.

|| Platelet function analysis-epinephrine.

¶ Prothrombin time.

∗∗ Activated partial thromboplastin time.

†† Odds ratio.

All patients who received revision surgery due to POSEH were recovered immediately and left no neurological sequelae.

## 4 Discussion

Though minimal invasive surgery is beneficial in many aspects, it still has inherent complications.^[1,2]^ Incomplete surgery was a main concern, however, the advent of BESS broke through the limited viewing and working angle.^[3–6,10–12]^ It allows extending the surgical indications of an endoscopic spine surgery up to the level of an open conventional decompression surgery. However, some pioneers have reported frequent development of POSEH.^[8,13]^ POSEH is the most common cause of neurological complications in a spine surgery.^[14]^ Unfortunately, the incidence, pathogenesis and risk factors of POSEH are not fully elucidated yet.^[15–19]^ Furthermore, we presumed that BESS itself can be a risk factor of POSEH. It is a rational conjecture to assume that more amount of fluid would be collected at epidural space after a fluid base endoscopic surgery. Clear fluid has homogenous signal on MRI, however, clotted hematoma is usually heterogenic. We can easily discriminate it from residual saline collection. And in the revision cases for hematoma evacuation, we did not find fluid collection over the hematoma mass. Kim et al. reported its risk factors in BESS as female sex, old age, preoperative anticoagulation medication and usage of intra-operative saline infusion pump.^[8]^ All others except for saline infusion pump are already reported as risk factors in the previous studies for conventional spine surgeries. We also used saline infusion pump in all BESS cases and cautiously agree with their results. While performing BESS, saline pressure makes the working space like as a joint arthroscopic surgery. We used saline infusion pump with the pressure of 50 mm Hg, higher than average venous pressure 40 mm Hg to prevent venous bleeding. So we assumed that the venous bleeding was masked during BESS. Therefore setting the infusion pump pressure less than 40 mm Hg would be recommended. And further study about the relationship between the pressure and POSEH is on the way in my institute. There are more assumptions. It is difficult to control the bleeding intraoperatively. Hemostasis of soft tissue and epidural bleeding can be done with a radiofrequency coagulator, however, bone bleeding is difficult to be controlled because covering of the bleeding surface with a bone wax is hampered by saline flow. Besides, compression of the operation site by tight suture of muscles and fascia is impossible. In our cohort, the ratio of spinal stenosis was greater in BESS than CSS. A spinal stenosis surgery usually demands bilateral laminectomy and bilateral yellow ligament removal that end up with greater exposure of bare cancellous bone surface and epidural vessels comparing to a HLD surgery. In the subgroup analysis of BESS, bilateral laminectomy was a risk factor of morphometric POSEH. We assumed that it has something to do with the difficulty of applying bone wax in BESS. Besides low platelet function was close to the significant level. Optimal platelet function is supposed to be more important in BESS because meticulous hemostasis is difficult in BESS. The subgroup analysis of CSS was not done because the number of POSEH was too small to have a meaningful result.

There were 10 patients who had a revision surgery for evacuation of the POSEH. The neurological deficit of all 2 patients of CSS was nG1 and they underwent the revision surgery under a general anesthesia. There were 6 of nG1, one of nG2 and one of nG3 in BESS. The patient with nG3 in BESS received revision surgery under general anesthesia because blood pressure was too high and prone position was intolerable due to severe leg pain and all others did it under local anesthesia with BESS technique again. There was no patient without neurological improvement by a revision surgery. All of them showed immediate improvement after a revision surgery and did not have neurological deficit at the time of discharge.

There were limitations in this study. Because it was a retrospective study, 2 groups were not completely homogenous. Especially, the ratio of stenosis was greater in BESS. But the risk factor analysis to the all subjects with multivariable analysis would offset the drawback. The anti-platelet drug medication was not considered. The 2 groups received operations at the different period of time. Although the operators were identical, the BESS subjects included early learning curve period cases. The sample size was not enough to verify statistical significance for our presumptions.

## 5 Conclusion

The incidence of clinical and morphologic POSEH was higher in BESS. In BESS, morphometric POSEH developed more frequently in bilateral laminectomy comparing to unilateral laminectomy and low platelet function gave considerable influence. We thought that it was related to the masking of epidural venous bleeding by saline pressure while doing a BESS. Besides, it seems that bleeding from the bone surface is the main reason why BESS is complicated by POSEH more frequently.

## Author contributions

**Conceptualization:** Dong Ki Ahn.

**Formal analysis:** Won Shik Shin.

**Investigation:** Dong Ki Ahn, Won Shik Shin.

**Methodology:** Jung soo Lee, San Kim, Jin Jung.

**Resources:** San Kim.

**Writing – original draft:** Jin Jung.

## Abbreviations

Abbreviations: BESS = biportal endoscopic spine surgery, CSS = conventional spine surgery, hG = hematoma grade, HLD = herniated lumbar disc, LSS = lumbar spinal stenosis, MRI = magnetic resonance imaging, nG = neurological grade, POSEH = postoperative spinal epidural hematoma.
